# Newborn Screening of X-Linked Adrenoleukodystrophy in Italy: Clinical and Biochemical Outcomes from a 4-Year Pilot Study

**DOI:** 10.3390/ijns11040084

**Published:** 2025-09-24

**Authors:** Eleonora Bonaventura, Fabio Bruschi, Luisella Alberti, Clara Antonello, Filippo Arrigoni, Marina Balestriero, Barbara Borsani, Laura Cappelletti, Elisa Cattaneo, Matilde Ferrario, Giulia Fiore, Maria Iascone, Giana Izzo, Simona Lucchi, Cecilia Parazzini, Michela Perrone Donnorso, Luigina Spaccini, Ylenia Vaia, Pierangelo Veggiotti, Elvira Verduci, Gianvincenzo Zuccotti, Cristina Cereda, Davide Tonduti, XALD-NBS Study Group

**Affiliations:** 1 Center for Diagnosis and Treatment of Leukodystrophies and Genetic Leukoencephalopathies (COALA), Vittore Buzzi Children’s Hospital, 20154 Milan, Italy; eleonora.bonaventura@asst-fbf-sacco.it (E.B.); fabio.bruschi@unimi.it (F.B.); luisella.alberti@asst-ovestmi.it (L.A.); clara.antonello@asst-fbf-sacco.it (C.A.); filippo.arrigoni@asst-fbf-sacco.it (F.A.); marina.balestriero@asst-fbf-sacco.it (M.B.); barbara.borsani@asst-fbf-sacco.it (B.B.); laura.cappelletti@asst-fbf-sacco.it (L.C.); elisa.cattaneo@asst-fbf-sacco.it (E.C.); matilde.ferrario@asst-fbf-sacco.it (M.F.); giulia.fiore@unimi.it (G.F.); giana.izzo@asst-fbf-sacco.it (G.I.); simona.lucchi@asst-fbf-sacco.it (S.L.); cecilia.parazzini@asst-fbf-sacco.it (C.P.); michelaperronedonnorso@gaslini.it (M.P.D.); luigina.spaccini@asst-fbf-sacco.it (L.S.); ylenia.vaia@unimi.it (Y.V.); elvira.verduci@unimi.it (E.V.); cristina.cereda@asst-fbf-sacco.it (C.C.); 2 Child Neurology Unit, Vittore Buzzi Children’s Hospital, 20154 Milan, Italy; pierangelo.veggiotti@unimi.it; 3 Department of Biomedical and Clinical Sciences, University of Milan, 20157 Milan, Italy; gianvincenzo.zuccotti@unimi.it; 4 Center of Functional Genomics and Rare diseases, Department of Pediatrics, Vittore Buzzi Children’s Hospital, 20154 Milan, Italy; 5 Pediatric Radiology and Neuroradiology Department, Vittore Buzzi Children’s Hospital, 20154 Milan, Italy; 6 Department of Pediatrics, Vittore Buzzi Children’s Hospital, University of Milan, 20154 Milan, Italy; 7 Clinical Genetics Unit, Department of Obstetrics and Gynecology, Vittore Buzzi Children’s Hospital, University of Milan, 20154 Milan, Italy; 8 Medical Genetics Laboratory, ASST Papa Giovanni XXIII, 24127 Bergamo, Italy; miascone@asst-pg23.it; 9 Metabolic Diseases Unit, Department of Pediatrics, Vittore Buzzi Children’s Hospital, University of Milan, 20154 Milan, Italy; 10 Department of Health Sciences, University of Milan, 20146 Milan, Italy

**Keywords:** X-linked adrenoleukodystrophy, ALD, NBS, newborn screening, Zellweger, ZSD

## Abstract

X-linked adrenoleukodystrophy (X-ALD) is the most common peroxisomal disorder, caused by mutations in the *ABCD1* gene. Early diagnosis is critical to manage adrenal insufficiency and cerebral forms of the disease. Since 2021, a pilot newborn screening (NBS) program for X-ALD has been launched in Lombardy, Italy. From September 2021 to June 2025, 138,116 newborns (≥37 weeks’ gestational age) were screened for elevated C26:0-lysophosphatidylcholine (C26:0-LPC) levels using a two-tier algorithm. Genetic testing was performed in non-negative cases. Males found to be *ABCD1* variant carriers were enrolled in multidisciplinary follow-up, including neurological, endocrinological, and nutritional assessments. Eleven individuals (six males, five females) carried pathogenic or likely pathogenic *ABCD1* variants. Three males were diagnosed with adrenal insufficiency and started hydrocortisone therapy between 1 and 2 years of age. Growth parameters were within normal range overall, but two children showed signs of stunting associated with poor dietary compliance. Additionally, three patients were diagnosed with Zellweger spectrum disorders (ZSDs). No patients affected with Aicardi-Goutières Syndrome were identified. Newborn screening for X-ALD in Italy is feasible and enables early detection and intervention. Biochemical markers and genetic analysis are reliable tools for identifying affected males and female carriers. Multidisciplinary management is essential to address medical and psychosocial challenges during follow-up.

## 1. Introduction

X-linked adrenoleukodystrophy (X-ALD) is the most common inherited peroxisomal disorder, resulting from mutations in the *ABCD1* gene, located on the X chromosome [1]. This gene encodes a peroxisomal membrane protein that is essential for transporting very long-chain fatty acids (VLCFAs) into peroxisomes, where they are broken down and eliminated [2].

A defect in *ABCD1* leads to the accumulation of VLCFAs in various tissues, particularly in the brain, spinal cord, and adrenal cortex. causing chronic inflammation, mitochondrial dysfunction from oxidative stress, and ultimately cell death [3]. Oxidative stress and impaired mitochondrial function are central factors in the pathogenesis of X-ALD. The accumulation of VLCFAs, particularly C26:0-lysophosphatidylcholine (C26:0-LPC), disrupts membrane structure and mitochondrial function and this leads to oxidative stress and lipid peroxidation, which can trigger ferroptosis and progressive neurodegeneration [4].

Variants in *ABCD1* can be associated with different clinical phenotypes; the primary manifestations seen in males with X-ALD include Addison’s disease (i.e., adrenal insufficiency), cerebral ALD (cALD), and adrenomyeloneuropathy (AMN), and they may occur individually or in various combinations [3,5]. Conversely, most females carrying pathogenic variants in *ABCD1* (up to 88%) develop a milder, later-onset form of AMN [6], while Addison’s disease or central nervous system involvement is rare (less than 1% of cases) [7]. At present, it is not possible to establish a clear genotype-phenotype correlation. However, recent lipidomic studies have identified new biomarkers for X-ALD, revealing a more pronounced increase in C26:0-LPC in patients with cALD and Addison disease, already evident before the onset of clinical signs [8].

Early diagnosis is crucial for the effective management of both cALD and Addison’s disease. Hematopoietic stem cell transplantation (HSCT) is the standard therapeutic approach for cALD and the sooner the transplant is performed, the more favorable the long-term outcome. Due to the significant risks and comorbidities associated with HSCT, transplantation is recommended only for patients in the early stages of active cerebral disease (Loes score < 10) and no neurological symptoms. Because white matter changes detectable on MRI typically precede clinical symptoms, early radiological detection through a periodic neuroradiological follow-up is essential, as it provides a window for intervening before neurological decline begins [9,10,11]. Gene therapy is also being explored as an alternative, particularly in cases where suitable allogeneic donors are unavailable, but it is not yet available in Europe [12].

The majority of males with ALD will eventually develop Addison’s disease, a condition that causes significant morbidity and mortality. Addison’s manifestations are often non-specific and may be easily overlooked or misdiagnosed, even in acutely ill individuals who were previously considered healthy. Therefore, early identification of *ABCD1* pathogenic variants is critical to regularly monitor adrenal hormone function and initiate hormone replacement therapy promptly when necessary [12].

Other therapeutic options for X-ALD include leriglitazone, a selective peroxisome proliferator-activated receptor gamma (PPARγ) agonist that helps restore energy balance and mitochondrial function. Recent clinical trials have shown it to be effective in both adult and paediatric cALD patients in preventing disease progression [13,14]. Dietary treatment aiming at reducing the accumulation of exogenous and endogenous VLCFA and reducing oxidative stress is controversial and the efficacy of a VLCFA-restricted diet therapy, together with the supplementation of nutraceuticals such as Aldixyl^®^, is still debated, although some evidence has been reported in the literature [15].

Recognizing the critical importance of early diagnosis in X-ALD, many countries have introduced newborn screening (NBS) programs over the past decade. These programs focus on measuring C26:0-LPC levels in dried blood spots (DBS). New York introduced this screening in 2013 [16] and by February 2016, X-ALD was added to the U.S. Recommended Uniform Screening Panel (RUSP). Since then, other states have followed suit [17,18,19,20,21,22,23,24,25,26]. In June 2021, the first X-ALD NBS pilot study started in Italy, supported by the Ministry of Health (GR-2019-12368701).

This study aims to present the results four years after the initiation of this pilot study.

## 2. Materials and Methods

From September 2021 to June 2025, 33 Neonatal Care Units in Lombardy region (Italy) participated in our pilot project, according to the algorithm previously published [27]. This project was led by a multidisciplinary team from Vittore Buzzi Children’s Hospital in Milan, Italy. The program also benefited from the expertise of two laboratories: the Regional Newborn Screening Referral Laboratory at Vittore Buzzi Children’s Hospital and the Medical Genetics Laboratory at Papa Giovanni XXIII Hospital in Bergamo. Two patients were genetically tested at the Medical Genetics Laboratory of the IRCCS Foundation Carlo Besta Neurological Institute in Milan, as genetic analyses of affected family members had previously been performed at this Center.

Genetic variants were classified as described in [27]. In cases of a variant of uncertain significance (VUS) in the *ABCD1* gene, patients were enrolled in the Grey Zone Project, an ALD Connect research initiative [28].

The study received centralized approval from the Ethics Committee of Milan Area 1 (2020/ST/395).

All infants born in Lombardy with a gestational age of ≥37 + 0 weeks, regardless of sex, were eligible for X-ALD NBS. Participation in the screening was voluntary and required informed consent signed by both parents. In cases of “non-negative” results from the first- and second-tier screening tests, an additional written consent specific to genetic testing was requested.

The newborn with non-negative screening and no symptoms underwent targeted genetic analysis as previously described, while the non-negative symptomatic infant underwent rapid trio-whole genome analysis, as recently described [27,29].

Regarding the methods used for sample collection, biochemical analysis, and gene sequencing, please refer to the details provided in the article describing the pilot project [27].

Patients with a confirmed genetic diagnosis were enrolled in a follow-up protocol and regularly monitored, according to international guidelines. The protocol also includes patients who are carriers of *ABCD1* variants of uncertain significance (VUS). The follow-up involves a multidisciplinary approach, including neuropsychiatric, neuromotor, neurocognitive, neuroradiological, endocrinological, nutritional and dietary assessments (for details, refer to [27]). During the biannual disease follow-up, patients also undergo a blood draw, and C26:0-LPC levels are monitored by DBS. Female carriers of *ABCD1* variants are not enrolled in the routine follow-up protocol. Instead, they undergo a single neurological consultation that includes genetic counselling for family screening. Following this, they are discharged with recommendations for neurological follow-up in adulthood and genetic counselling for any future pregnancies.

In reference to the nutritional follow-up, more detailed data are presented here compared to those reported in the previous study. During routine inpatient visits, all patients underwent full anthropometric assessment from specialized dietitians or nutritionists, to monitor growth, adherence to prescribed dietary regimen and the safety (absence of side effects) of these regimens. Weight and height were measured recumbent by means of an electrical column scale (Soehnle 7725), and an infantometer (Seca 416), respectively. Weight z-score, height z-score and weight-for-length/height were assessed according to World Health Organization growth standards [30]. Children under 2 years of age were classified as wasted in case of weight-for-length < −2 z-scores, and at risk of wasting between −2 and −1 z-scores [30]. Stunting was defined in case of length/height-for-age < −2 z-scores, while the risk for stunting between −2 and −1 z-scores according to WHO growth reference [30]. Head circumference was assessed with measuring tape (Seca 201). Also body mass index (BMI) was calculated as the ratio between weight (kg) and height squared (m^2^). BMI-for-age was also assessed according to WHO z-scores [30,31]. Mid-upper arm circumferences (MUAC) were measured with measuring tape (Seca 201), then MUAC z-score (MUACz) was calculated [32]. Skinfolds tricipital (TSF), bicipital, subscapular, and supra-iliac were measured three times (Holtain 610) and recorded to the closest 0.2 mm. The z-scores for tricipital skinfold (TSFz) were calculated [33].

**Figure 1:**
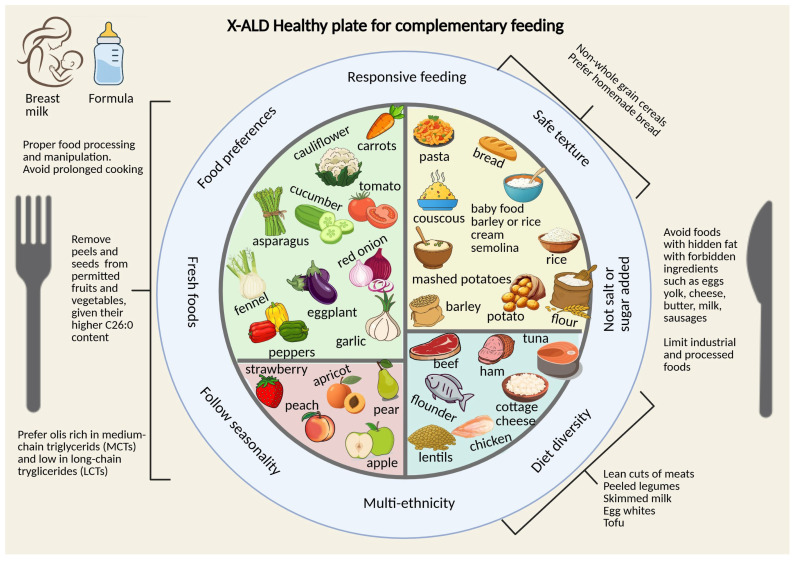
X-ALD healthy plate for complementary feeding. Allowed protein-rich foods according to C26:0 and total fatty acids content: Meat alternatives (beef lean cuts, chicken meat without skin, horse meat, ham (cooked and raw) with fat completely trimmed, turkey breast, pork meat lean cuts); Fish alternatives (flat fish, flounder, monkfish, octopus, perch, pike, sole, squid and cuttlefish (no tentacles), seabream, turbot, tuna fish canned natural); Egg white; Legumes (lentils peeled, white beans peeled, tofu); Milk-derived products (cottage cheese 1% fat, yogurt from skimmed milk plain 0% fat, skimmed milk 0% fat).

A pediatric nutritional visit was scheduled within 3 months of birth, and within 6 months before starting complementary feeding, a dietary assessment and nutritional counselling were offered by dietitians and nutritionists. The family was instructed on how to adopt a diet naturally low in VLCFA content from complementary feeding onwards. Given the crucial role of dietary lipids for growth and neurodevelopment, no dietary restrictions were applied to the daily intake of lipids up until 24 months of age. Specific information on VLCFA content in foods and handling strategies to reduce its amount was provided to caregivers. To deliver dietary counselling to patients and caregivers, we specifically developed the X-ALD Healthy Plate for complementary feeding (see Figure 1).

## 3. Results

### Neonatal Biochemical Findings

From September 2021 to June 2025, among the 237,548 live-born infants with a gestational age of over 37 weeks, 138,116 (51.30% or 70,854 males, 48.69% or 67,255 females, 0.01% or seven unknown) consented to participate in the X-ALD newborn screening pilot study (XALD-NBS).

In total, 298 neonates showed a “non-negative” result for the C26:0-LPC metabolite in the first-tier assay (cut-off: <0.5 µmol/L). Among these, fourteen remained “non-negative” upon second-tier testing, which relied on a cut-off <0.1 µmol/L; of these 14 neonates, eight were males and six females.

C26:0-LPC results at first and second tier tests are reported in Table 1.

## 4. X-ALD Patients

Eleven patients were found to carry variants in the *ABCD1* gene (six males and five females). All of these were classified as pathogenic or likely pathogenic; only one was classified as a VUS (Phe265Tyr). To assess the pathogenicity of the variant, the patient was included in the Grey Zone Project [28]. C26:0-LPC analysis on DBS was centrally analysed at the Laboratory Genetic Metabolic Diseases at Amsterdam UMC, confirming a value significantly above the control range (285 nmol/L; normal values 29–72 nmol/L). As a result, the patient was enrolled in the follow-up program under the same protocol applied to the other identified cases.

*ABCD1* variants were found to be maternally inherited in all eleven patients. Mothers were advised to begin neurological follow-up at a specialized adult centre.

By reconstructing the family trees of the newborns who tested positive, extended genetic counselling was provided to family members for early disease detection. Among those who underwent genetic testing, just two additional carriers of an *ABCD1* variant were identified (the older sister and a pre-symptomatic adult uncle of a female carrier). In three cases, one male and two females, the family history revealed previously diagnosed cases of the disease.

In all males, regular follow-up was started according to the protocol we previously described [27] and they were all neurologically asymptomatic at the last follow-up visit.

**Figure 2:**
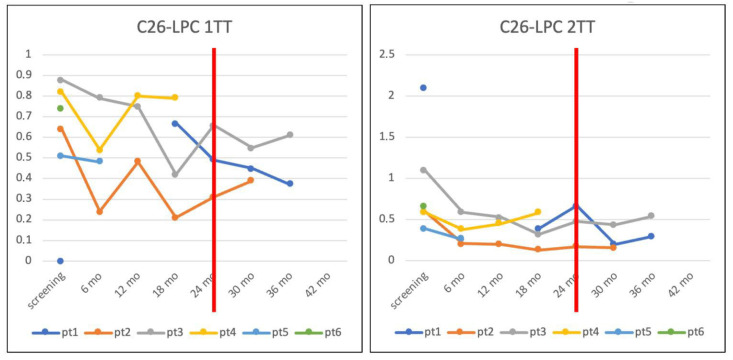
Plasma C26:0-LPC concentration trends in patients undergoing clinical follow-up. C26:0-LPC concentrations over time. Note the initial decline in the first six months, followed by stabilization. Red lines indicate the start of dietary treatment, which did not normalize metabolite levels. Legend: Pt = patient, mo = months, 1TT = first-tier test, 2TT = second-tier test, LPC = lysophosphatidylcholine.

Table 2 illustrates the longitudinal profile of plasma C26:0-LPC concentrations, which were measured at 6-month intervals during the protocol-defined follow-up. Values have also been graphed in Figure 2 and as illustrated, an initial decline within the first six months of life is evident, followed by largely stable metabolite concentrations irrespective of the clinical phenotype. The initiation of dietary treatment with a very long-chain fatty acid (VLCFA)-restricted diet and a combination of Lorenzo’s Oil (LO) and antioxidant compounds (Aldixyl^®^, Aldixyl^®^ OILife) has not yet led to normalization of C26:0-LPC levels. 

### 4.1. Endocrinological Issues

Since the launch of our pilot screening program in 2021, all male patients diagnosed with X-ALD have undergone paediatric endocrinological evaluations in accordance with our protocol [27] and international guidelines [12]. AI was identified in three patients (Table 3): two with complete and one with partial AI, diagnosed via ACTH stimulation testing (125 µg intravenous cosyntropin). In patients with confirmed AI or those older than two years, additional assessments of plasma renin activity, aldosterone, and serum electrolytes revealed no evidence of mineralocorticoid deficiency. Notably, none of these patients displayed clinical signs or symptoms of AI at the time of diagnosis or during early stress episodes.

Patients with complete AI initiated hydrocortisone therapy at 8–10 mg/m^2^/day, divided into three doses. The patient with partial AI began treatment at 7 mg/m^2^/day in two doses. Granule formulations allowed for precise dose titration in these young children.

Patient 1 presented at 12 months with elevated ACTH (153.8 ng/L) and normal cortisol (139 µg/L). Given his stable clinical condition, a watchful waiting strategy was adopted, following current guidelines, with quarterly monitoring of basal hormone levels. ACTH levels progressively increased (171 → 240 → 239 ng/L), while cortisol consistently remained above 100 µg/L. Daily glucocorticoid therapy was initiated at 21 months following an ACTH stimulation test.

Patient 2 exhibited increasing but fluctuating ACTH levels, with cortisol levels consistently above 100 µg/L by 9 months of age. By age two, ACTH remained above 100 ng/L in all assessments. The patient remains asymptomatic, and ACTH stimulation testing is planned soon.

Patient 3 underwent ACTH stimulation testing at 3 months due to elevated ACTH (88 ng/L) and low cortisol (45 µg/L). Given this X-ALD diagnosis and concurrent cardiac follow-up for aortic coarctation (post-neonatal surgery, with reintervention planned at 4 months), a cautious approach was taken. The ACTH test showed an adequate cortisol response (ACTH 223 ng/L; cortisol 128 → 211 µg/L), and perioperative stress-dose steroids were recommended. However, ACTH levels continued to rise over the following months, prompting initiation of daily glucocorticoid therapy at 13 months, as with Patient 1.

Patient 4 demonstrated fluctuating ACTH values (<100 ng/L) and cortisol levels ranging between 60 and 130 µg/L during the first year of life. At 18 months, ACTH was 179 ng/L and cortisol 83 µg/L. ACTH stimulation testing confirmed partial AI, and stress-dose steroids were recommended. After discussion with the family, low-dose daily glucocorticoid therapy was started to reduce caregiver anxiety about managing potential adrenal crises in the absence of a baseline treatment.

The remaining two male patients with X-ALD (Patients 5 and 6, currently 9 and 8 months old, respectively) have demonstrated normal adrenal function during ongoing quarterly follow-ups.

All six families received an Adrenal Crisis Emergency Letter containing individualized information and detailed recommendations for diagnostic and therapeutic interventions in the event of an adrenal crisis, to be presented in emergency pediatric care settings.

### 4.2. Nutritional Assessment

All children underwent the first pediatric nutritional visits within 3 months of life (0.34 ± 0.12 years of age at first visit). During the first visit, information on breastfeeding practices was collected. Half of the cohort (50%) was exclusively breastfed until the start of complementary feeding, while 50% were formula-fed or started formula feeding during the first 6 months of life.

Before starting complementary feeding, all patients underwent a nutritional assessment (0.56 ± 0.14 years of age). At baseline, the mean weight-for-age SDS was −0.13 ± 0.37, length/height-for-length SDS was 0.19 ± 0.77 and weight-for-length/height was −0.27 ± 0.82. One subject at baseline was at risk of wasting (weight-for-length −1.79 SDS). Regarding the anthropometric assessment, MUAC SDS and TSF SDS at baseline were within normal ranges and equal to 0.01 ± 0.90 and −0.13 ± 1.05, respectively.

Growth was monitored during the 2-year follow-up with at least one annual visit. Mean weight-for-length SDS were 0.45 ± 0.76 at T1 and 0.61 ± 0.28 at T2, respectively. All subjects show proper trends towards weight gain, and no subject was classified as wasted at either T1 or T2. On the contrary, we observed a trend toward a decrease in mean length/height-for-age SDS at T1 and T2 (−0.57 ± 1.06 and −0.77 ± 0.56, respectively). Specifically, three patients at T1 and two patients at T2 were classified as “at risk of stunting”.

In the sample of subjects enrolled, there is reported difficulty in following the nutritional intervention. Interestingly 50% were classified as non-compliant and within this category are the two children at risk of stunting. The results are summarized in Table 4.

## 5. Non-ALD Patients Detected Through NBS

Out of 14 patients who tested non-negative at the second-tier test, three newborns were diagnosed with Zellweger spectrum disorders (ZSDs) and were all symptomatic at birth.

One male patient (Pt12) was born to consanguineous parents with no reported family history of neurological disorders. Pregnancy and delivery were uneventful. Generalized hypotonia was noted at birth. Brain MRI performed at 16 days of life demonstrated bilateral temporo-insulo-fronto-parietal polymicrogyria and cerebellar subcortical heterotopias. Targeted WGS revealed a compound heterozygous deletion in the *ACOX1* gene, consisting of a complete gene deletion of maternal origin and an exon 3 deletion of paternal origin. The clinical course was characterized by developmental delay, global hypotonia, atonic-myoclonic epilepsy, bilateral sensorineural deafness, mild retinopathy, and adrenal insufficiency.

One male patient (Pt13) was born to consanguineous parents, with a family history of epilepsy, febrile seizures, and neonatal seizures. At birth, the patient presented with respiratory distress, facial dysmorphisms, generalized hypotonia, epileptic seizures, optic nerve hypoplasia, feeding difficulties, central apnoea and renal cysts. Brain MRI revealed diffuse structural abnormalities, more prominent in the perisylvian regions, and delayed myelination for age. The clinical course was rapidly progressive, culminating in death from cardiopulmonary failure and recurrent seizures at 2 months of life. Genetic testing identified a homozygous c.193G>C (p.Gly65Ser) variant in the *PEX10* gene.

The third ZSD female patient (Pt14) was found to carry a homozygous duplication in *PEX26* [Chr22(GRCh38): g.18079213_18080053dup]. The clinical presentation is extensively described in Visani et al. [34].

No patients with variants in genes related to Aicardi–Goutières syndrome were identified.

## 6. Discussion

We reported the results of a 4-year pilot study on XALD-NBS conducted between September 2021 and June 2025. Our results confirm that the integration of X-linked adrenoleukodystrophy (X-ALD) into newborn screening programs is feasible and enables early diagnosis and prompt disease surveillance, thereby allowing for the timely implementation of therapeutic interventions.

In fact, early diagnosis is crucial for initiating rigorous endocrinological monitoring and management of Addison’s disease, a prevalent and potentially life-threatening manifestation of X-ALD. Moreover, considering the established efficacy of hematopoietic stem cell transplantation (HSCT) in halting the progression of cerebral demyelinating lesions (cALD), when performed during the earlier stages of the disease, the integration of X-ALD into newborn screening becomes fundamental to significantly change the natural history of this disorder in affected individuals.

C26:0-lysophosphatidylcholine is a confirmed sensitive and specific marker for X-linked adrenoleukodystrophy and Zellweger spectrum disorders diagnosis through newborn screening programs [8]. On the other hand, although C26:0-LPC has previously been reported as a metabolite suitable for identifying patients with Aicardi–Goutières syndrome (AGS) through NBS programs, we did not identify any such patients in our study. It is important to recall, however, that those prior observations were based on first-tier test measurements, with the second-tier test failing to detect these patients [35,36]. It is, therefore, plausible that the two-tiered approach of C26:0-LPC testing for X-ALD may not be the most sensitive for diagnosing AGS via newborn screening.

**Figure 3:**
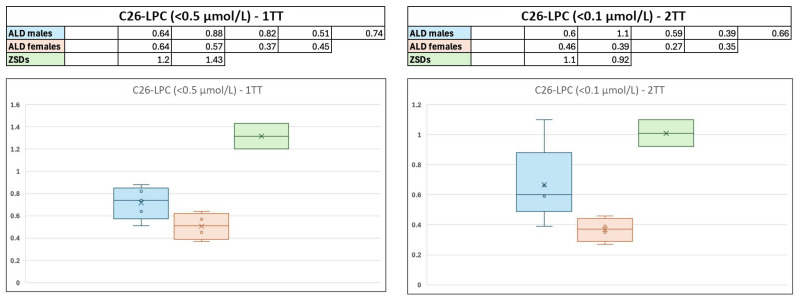
Box and whisker plots illustrating neonatal C26:0-LPC concentrations. Data are presented for male and female *ABCD1* variant carriers and ZSD patients. Legend: ALD = adrenoleukodystrophy, ZSDs = Zellweger Spectrum Disorders, 1TT = first-tier test, 2TT = second-tier test, LPC = lysophosphatidylcholine.

Since the launch of our X-ALD screening program, experimental conditions have evolved significantly. We have adjusted cutoff values and enhanced the accuracy of reference ranges for identifying X-ALD patients. Measuring acylcarnitines (C20 to C26) and phosphatidylcholines initially presented numerous technical challenges, particularly concerning specificity during method development and the first few months of operation. Consequently, the cut-off for C26-LPC was modified from an initial 0.65 µM to 0.5 µM in the early stages of the program. This issue was not observed with the second-tier test, likely due to the distinct characteristics of the first-tier test’s flow injection compared to the chromatographic separation method used in the second tier. As a result, the reported concentrations for the very first cases identified may be subject to this analytical bias, which complicates the direct comparison of these initial concentrations with those obtained subsequently. Based on the published literature [23], which has shown that the first-tier test and second-tier test clearly differentiated between carriers of *ABCD1* variants and Zellweger Spectrum Disorder (ZSD) patients, we sought to determine if the values from our cohort exhibited a similar discriminatory ability. To ensure the accuracy of our analysis, we excluded the initial concentrations from early *ABCD1* cases (one male, one female; blank cells) and the concentration from the first identified *ACOX1* patient (blank cell), due to potential analytical bias and significant biochemical overlap with *ABCD1* dysfunction, respectively. Our findings, as shown in Figure 3, appear to be consistent with the observations reported in the literature. In fact, C26:0-LPC levels at both tiers were markedly higher in patients with ZSDs compared to the intermediate levels found in males with X-ALD, and to slightly abnormal levels observed in females. It is of note that among the three ZSD patients, the two with gene variants associated with peroxisomal biogenesis defects (*PEX10*, *PEX26*) show distinctly higher values compared to X-ALD patients. In contrast, the patient with an *ACOX1* variant presents less marked differences, possibly due to greater biochemical similarity with *ABCD1* disfunction as the gene is linked to dysfunction of the peroxisomal acyl-coenzyme A oxidase 1, which catalyses the first step of VLCFA β-oxidation. Unlike our findings, the C26:0-LPC values between male and female *ABCD1* variant carriers in the cohorts of the previous studies [23] were overlapping and did not allow for a distinction; a larger series of patients will be needed to better clarify this point.

Monitoring plasma C26:0-LPC concentrations during patients’ biannual follow-up revealed an initial decline within the first six months of life, followed by a largely stable trend in metabolite concentrations, irrespective of the clinical phenotype (Addison-only versus asymptomatic). As already pointed out in the literature, this is likely because newborns have a greater red blood cell mass compared to adults [37] in the bloodstream and C26:0-LPC is mainly localized within the membranes of erythrocytes [38,39]. Three patients reach the age of 24 months, when dietary treatment with a VLCFA-restricted diet and a mixture of Lorenzo’s Oil (LO) and antioxidant compounds is usually started (Aldixyl^®^, Aldixyl^®^ OILife). Normalization of C26:0-LPC levels has not been reached so far; it is, however, important to note that adherence to the treatment was not optimal. A recent study suggests that nutritional counselling and a specific diet, particularly a Mediterranean one, can reduce C26:0-LPC plasma concentrations [15]. Our sample size does not permit drawing conclusions in support or against this hypothesis, but larger numbers and longer follow-up will be available in the near future.

As mentioned, within the context of the pilot project, proband-only whole-genome sequencing (WGS) targeted to *ABCD1* was adopted for asymptomatic patients following non-negative first- and second-tier testing. This offers the advantage of a rapid and comprehensive genetic assessment, but also requires substantial resources and costs. Given that most pathogenic *ABCD1* variants are single-nucleotide variants (SNVs) and less than 4% are rearrangements [40], a more cost-effective approach could be considered. We suggest that a first-level genetic test using traditional targeted methods be performed on non-symptomatic newborns with non-negative screening results. WGS could then be reserved for cases that are inconclusive after this initial testing or for newborns who are symptomatic at birth.

As part of our pilot project, we decided to include females in the screening program. This choice introduces certain challenges, as the condition typically presents earlier and more severely in males, whereas females tend to develop only mild symptoms at a later stage. Moreover, unlike males, who may benefit from treatments for cALD and AI, no such effective and recognized therapies currently exist for females. Nevertheless, identifying female carriers has allowed us to analyse family pedigrees and proceed with genetic testing of potentially affected males who were not screened at birth. In addition, the identification of female newborns with *ABCD1* variants facilitated the subsequent identification of their carrier mothers and referred them for neurological evaluation and long-term follow-up.

Another potential advantage of including females in newborn screening programs is the opportunity to provide them with appropriate prenatal counseling once they reach reproductive age. Endocrinological findings in our study align with findings from other newborn screening cohorts for X-ALD. AI was identified in three out of six male patients with X-ALD, confirming the high prevalence of adrenal involvement in this condition [41]. A fourth patient is showing progressive hormonal changes suggestive of evolving AI, while the two remaining patients, currently 8 and 9 months old, are still under 1 year of age, a timeframe in which AI may not yet be apparent. Our findings confirm the possibility of early identification of AI. All affected patients were diagnosed and started glucocorticoid therapy between 1 and 2 years of age. The surveillance program also enabled detection of abnormal ACTH and cortisol values months before clinical AI emerged: Patients 1 and 2 showed biochemical changes 9 and 10 months, respectively, prior to diagnosis. Notably, none of the patients exhibited clinical symptoms at the time of diagnosis, underscoring the silent progression of adrenal dysfunction in X-ALD and the critical role of routine biochemical monitoring. This is particularly relevant in Patient 2, whose underlying cardiac disease could have been life-threatening in the event of an unrecognized adrenal crisis. Prompt initiation of hormone replacement therapy in asymptomatic patients based on elevated ACTH and/or low cortisol follows best practices and current guidelines [12]. All patients were treated with physiological doses of hydrocortisone (8–10 mg/m^2^/day, divided into 2–3 doses), in line with pediatric recommendations to avoid both under- and overtreatment. Importantly, normalization of ACTH is not a therapeutic goal, as adrenal tissue is relatively insensitive to ACTH stimulation; attempting to normalize ACTH would require supraphysiological dosing, risking overtreatment [42]. No cases of mineralocorticoid deficiency were observed, consistent with previous reports indicating that aldosterone deficiency is rare in X-ALD-related AI. The distribution of an Adrenal Crisis Care Letter to all families represented a key emergency preparedness measure, aimed at minimizing delays in recognition and treatment of adrenal crises. This proactive strategy, supported by patient advocacy groups, is especially important for emergency care settings where knowledge of rare metabolic or endocrine disorders may be limited.

In our study, anthropometric measurements were generally within normal ranges, although some cases showed risk of stunting and wasting. Wasting, when present at baseline, was promptly addressed during follow-up. In contrast, stunting tended to develop over time, particularly in patients who were also classified as non-adherent to dietary interventions. Continuous growth monitoring and longitudinal follow-up are essential to optimize growth in patients with X-ALD. The reported nutritional data constitute a baseline evaluation, which was deemed essential to establish a reference point at the initiation of the dietary treatment. This assessment was performed to specifically evaluate the effects of a VLCFA-restricted diet, combined with the use of antioxidant supplements as aforementioned, on patient growth, independent of their potential efficacy on the disease pathogenic mechanisms. Future studies should investigate the extent to which adherence to nutritional interventions influences linear growth trajectories.

## 7. Conclusions

Our findings contribute to the growing body of evidence supporting the inclusion of X-ALD in newborn screening (NBS) programs. International experience has demonstrated the feasibility and clinical utility of early screening, allowing for timely diagnosis and interventions that can prevent adrenal crises and enable early treatment of cerebral forms [43]. However, the implementation of NBS for X-ALD remains a subject of debate due to several practical and ethical concerns. Certainly, genetic analysis presents major challenges. Through screening, *ABCD1* variants of uncertain significance may also be identified, complicating both diagnosis and clinical management. Furthermore, the weak genotype–phenotype correlation in X-ALD limits the ability to predict disease severity or progression based on genetic data alone. Throughout the surveillance program, we observed varying degrees of anxiety among families, primarily driven by uncertainty surrounding the clinical course of X-ALD in their children. The involvement of a multidisciplinary team has proven essential in improving compliance and reducing stress related to ongoing monitoring. Notably, no participants have discontinued follow-up to date. We acknowledge that, compared to other diseases included in newborn screening programs, the inability to predict the timing and nature of symptom onset represents a significant limitation. Nevertheless, X-ALD NBS aims at identifying not only cALD, but it is also critical to prevent a sudden and severe adrenal crisis. Furthermore, from approximately two years of age, there is a substantial risk of developing the cerebral form of the disease. Since timely intervention can significantly alter the natural history of these manifestations, we do think that NBS and close longitudinal follow-up from the earliest months of life are essential to improve patient outcomes. Another significant challenge, requiring careful consideration of the cost–benefit balance and associated ethical considerations, is the identification of asymptomatic female carriers that may also carry the risk of psychological distress, stigmatization, and potential overmedicalization, particularly in the absence of clearly actionable interventions. These challenges underscore the importance of careful program design, ongoing family support, and continued international collaboration to harmonize diagnostic criteria, follow-up protocols, and ethical guidelines for X-ALD screening and management.

## Figures and Tables

**Table 1:** Comprehensive clinical, genetic, biochemical data of the study cohort. The table includes data from patients affected by X-ALD, female carriers of *ABCD1* variants, and individuals diagnosed with Zellweger spectrum disorders (ZSD). For each subject, the following data are reported: sex, age at last follow-up (y,m), genetic variant, C26:0-lysophosphatidylcholine concentrations at birth, neurological profile, and endocrine findings. Legend: Pt = patient, y m = years months, AI = adrenal insufficiency, LPC = lysophosphatidylcholine, 1TT = first tier test, 2TT = second tier test, DBS = dried blood spot.

	Demographics		Genetics				C26:0-LPC at Birth (DBS)		Neurology		Endocrinology	
Patient	Sex	Age at Last FU (y,mo)	Gene	Variant	Aminoacidic Change	Pathogenicity	1TT (µmol/L)	2TT (µmol/L)	Clinical Picture at Last Follow-Up	Brain MRI at Last Follow-Up	Clinical Picture at Last Follow-Up	Adrenal Crisis
Pt1	Male	3y 5mo	*ABCD1*	c.293C>T	p.Ser98Leu	pathogenetic	0.77	2.09	Normal	Normal	Addison only	0
Pt2	Male	3y	*ABCD1*	c.488G>A	p.Arg163His	pathogenetic	0.64	0.60	Normal	Normal	Asymptomatic (partial AI)	0
Pt3	Male	3y	*ABCD1*	c.1628del	p.Pro543Fs	pathogenetic	0.88	1.10	Normal	Normal	Addison only	0
Pt4	Male	1y 6mo	*ABCD1*	c.893G>T	p.Gly298Val	likely pathogenetic	0.82	0.59	Normal	Normal	Addison only	0
Pt5	Male	1y	*ABCD1*	c.794T>A	p.Phe265Tyr	likely pathogenetic	0.51	0.39	Normal	not performed as per protocol	Asymptomatic	0
Pt6	Male	0y 6mo	*ABCD1*	c.1687dup	p.Val563Glyfs*38	likely pathogenetic	0.74	0.66	Normal	not performed as per protocol	Asymptomatic	0
Pt7	Female	0y 1mo	*ABCD1*	c.1036A>G	p.Met346Val	likely pathogenetic	0.61	0.51	not evaluated as per protocol	not performed as per protocol	not performed as per protocol	not applicable
Pt8	Female	0y 1mo	*ABCD1*	c.1850G>A	p.Arg617His	likely pathogenetic	0.64	0.46	not evaluated as per protocol	not performed as per protocol	not performed as per protocol	not applicable
Pt9	Female	0y 1mo	*ABCD1*	c.532C>T	p.Gln178*	likely pathogenetic	0.57	0.39	not evaluated as per protocol	not performed as per protocol	not performed as per protocol	not applicable
Pt10	Female	0y 1mo	*ABCD1*	c.293C>T	p.Ser98Leu	pathogenetic	0.37	0.27	not evaluated as per protocol	not performed as per protocol	not performed as per protocol	not applicable
Pt11	Female	0y 1mo	*ABCD1*	c.270_279dup	p.Leu94Aspfs*104	pathogenic	0.45	0.35	not evaluated as per protocol	not performed as per protocol	not performed as per protocol	not applicable
Pt12	Male	2y 1mo	*ACOX1*	g.75829529_76012979del (maternal origin); g.75958635_75961120del–c.270-735_431-1059del (paternal origin)		pathogenic; pathogenic	0.79	0.56	Pathological at birth (see text)	leukodystrophy, cortical malformations	Addison disease	0
Pt13	Male	0y 2mo (died)	*PEX10*	c.193G>A (homozygous)	p.Gly65Ser; p.Gly65Ser	likely pathogenetic	1.20	1.10	Died, Pathological at birth (see text)	leukodystrophy, cortical malformations	not tested	0
Pt14	Female	0y 2mo (died)	*PEX26*	c.230+607_371+39dup (homozygous)		likely pathogenetic	1.43	0.92	Died, Pathological at birth (see text)	leukodystrophy, cortical malformations	not tested	0

**Table 2:** Longitudinal profile of plasma C26:0-LPC concentrations in ALD male patients. The table presents C26:0-lysophosphatidylcholine levels at the first and second tier tests, measured at birth and during the six-month follow-up evaluations in all male patients diagnosed with X-linked adrenoleukodystrophy. Legend: Pt = patient, mo = months, 1TT = first-tier test, 2TT = second-tier test.

	C26-LPC (<0.5 µmol/L)—1TT						
Patients	Screening	6th Month	12th Month	18th Month	24th Month	30th Month	36th Month
Pt1	0.77			0.67	0.49	0.45	0.37
Pt2	0.64	0.24	0.48	0.21	0.31	0.39	
Pt3	0.88	0.79	0.75	0.42	0.66	0.55	0.61
Pt4	0.82	0.54	0.80	0.79			
Pt5	0.51	0.48					
Pt6	0.74						
	C26-LPC (<0.1 µmol/L)—2TT						
Patients	Screening	6th Month	12th Month	18th Month	24th Month	30th Month	36th Month
Pt1	2.09			0.39	0.67	0.20	0.30
Pt2	0.60	0.21	0.20	0.13	0.17	0.16	
Pt3	1.10	0.59	0.53	0.32	0.48	0.44	0.54
Pt4	0.59	0.38	0.45	0.58			
Pt5	0.39	0.26					
Pt6	0.66						

**Table 3:** Endocrinological features of X-ALD male patients identified by newborn screening and diagnosed with AI. This table provides a detailed clinical and biochemical characterization of male patients with X-linked adrenoleukodystrophy (X-ALD) who developed Addison’s disease. Legend: Pt = patient, y m = years months, X-ALD = X-linked Adrenoleukodystrophy, ACTH = Adrenocorticotropic hormone, HC = hydrocortisone, Na = sodium, K = potassium.

Patients	Pt 1	Pt 3	Pt 4
	Addison’s Disease	Addison’s Disease	Partial AI
Birth date	2021	2022	2023
Age at onset of ACTH abnormal values	12 mo	3 mo	1 y 6 mo
Age at start hormonal treatment	1y 9mo	1y 1mo	1y 6mo
	(21 months)	(13 months)	(18 months)
ACTH (ng/L)	348	546	586
Cortisol (mcg/L)	104	84	127
Cortisol peak 60 min (mcg/L)	102	81	159
Fasting blood glucose (mg/dL)	81	74	70
Na/K (mmol/L)	134/4.5	138/4.1	139/4.7
Signs/symptoms	none	none	none
Glucocorticoid replacement therapy	HC granules	HC granules/tablets	HC granules
	8.6 mg/m^2^/day	9 mg/m^2^/day	7 mg/m^2^/day

**Table 4:** Growth and anthropometric parameters of the enrolled subjects at each time point (T0, T1, T2) during nutrition assessment visits. The table shows the mean anthropometric and growth parameters recorded in male patients with X-ALD at birth and during the six-month follow-up visits. Legend: BMI = body mass index, MUAC = Mid-upper arm circumference, AMA = arm muscular area, AFA = and arm fat area, WHO = World Health Organization, SD = standard deviation.

	T0		T1		T2	
	Mean	SD	Mean	SD	Mean	SD
Age	0.56	0.14	1.32	0.29	2.24	0.49
Weight (kg)	8.02	0.65	10.52	0.54	12.73	0.88
Weight-for-age SDS WHO	−0.13	0.37	0.05	0.36	0.04	0.34
Length/height (cm)	68.92	1.72	78.34	3.24	86.70	4.46
Length/height-for-age SDS WHO	0.19	0.77	−0.57	1.06	−0.77	0.56
Weight-for-length SDS WHO	−0.27	0.82	0.45	0.76	0.61	0.28
BMI (kg/m^2^)	16.87	1.09	17.18	1.31	16.85	0.53
BMI-for-age SDS WHO	−0.31	0.82	0.56	0.98	0.75	0.31
MUAC (cm)	14.38	1.11	15.25	0.50	16.28	0.93
MUAC SDS WHO	0.01	0.90	0.37	0.33	0.76	0.80
Skinfold tricipital	8.85	1.42	8.20	1.12	7.50	2.08
Skinfold tricipital SDS WHO	−0.13	1.05	0.22	0.78	−0.32	1.46
AMA%	65.15	3.80	69.13	3.52	73.28	6.71
AFA%	34.85	3.80	30.88	3.52	26.73	6.71

## Data Availability

Upon a motivated request, the corresponding author can provide access to the data supporting this study’s findings.
